# Effects of summer internship and follow-up distance mentoring programs on middle and high school student perceptions and interest in health careers

**DOI:** 10.1186/s12909-018-1205-3

**Published:** 2018-05-02

**Authors:** Emma Fernandez-Repollet, Craig Locatis, Wilfredo E. De Jesus-Monge, Richard Maisiak, Wei-Li Liu

**Affiliations:** 1 Center for Collaborative Research in Health Disparities, University of Puerto Rico Medical Campus, San Juan, PR USA; 2 National Library of Medicine/National Institutes of Health, Bethesda, MD 20894 USA; 3 School of Medicine, University of Alabama at Birmingham, Birmingham, AL USA

**Keywords:** Health career education, Distance mentoring, Health disparities, Minority students

## Abstract

**Background:**

Minorities are underrepresented in health professions and efforts to recruit minority students into health careers are considered a way to reduce health disparities. There is little research about the effectiveness of these programs, other than satisfaction. This study aimed to measure program effects on student understanding of and interest in health careers.

**Methods:**

Students took a career interest inventory, completed a scale measuring their self-reported understanding and interest in health careers, and wrote essays about health careers before and after completing a 1 week on campus internship on health careers and after a 9 month follow up distance mentoring program where they continued to interact with university faculty by videoconference about career options. Changes in inventory, scale, and essay scores were analyzed for changes over time using Wilcoxon and Mann-Whitney tests.

**Results:**

Inventory scores were unchanged over time, but scale and essay scores trended upward significantly post internship and mentoring.

**Conclusion:**

Health career education and mentoring programs can positively affect student knowledge of health careers and their attitudes about them. The study’s methods extend measures of program impact beyond satisfaction.

## Background

Minorities are under-represented in health professions and there are ongoing efforts to motivate minority middle and high school students to pursue health science careers. Very little is known about the effects of career education programs and there are few measures of program impact other than student satisfaction. The effects of a health career education and mentoring program for middle and high school students are reported in this article using measures of student career knowledge and choice. A rationale for minority health career programs is provided. Existing health career education research and the difficulties of measuring career interventions are discussed. The program and its evaluation methodology are described and results are presented. Implications for the development of future programs and for further research are examined. Three research questions were addressed: 1) Can short term internship and workshop programs affect student knowledge and interest in health careers? 2) Can longer term follow up mentoring programs add value and show continued knowledge and interest improvement? 3) What is the efficacy of different ways of measuring the impact of health career programs that go beyond ratings of satisfaction?

There are increasing efforts to recruit minorities into health professions. While African Americans, Hispanics, and American Indians together compose 25 % of the population, they comprise 9 % of nurses, 6 % of physicians, and 5 % of dentists [1]. Since minority health professionals are more likely to serve minority and underserved populations, the imbalance contributes to health care disparities [1, 2]. Career education and counseling research reviews and meta-analyses generally show positive outcomes [3, 4]. There are several career education objectives, including behavioral, motivational and attitudinal change; improved knowledge and skill; participation in post-secondary education; retention and achievement; job placement; and improved earnings [5]. There is evidence supporting aspects of each objective, but a theme common to all is that these outcomes improve when education and career choices are compatible with a student’s personality and interests [5]. Extensive individual counseling is considered the most effective career guidance intervention, but it is also the most expensive. Consequently, the use of computer-based and online resources combined with personal guidance may be the most practical approach [5].

Most health career research concerns graduate professional education. Literature searches of Scopus, Science Direct, and PubMed, identified only a handful of studies related to high school students and minorities. One study of a small, hospital-based mentoring program for African American high school students reported 92% attended college and 87% enrolled in health science degree programs [6]. Another similar hospital and university minority mentoring program that expanded from serving a small, elite group of students to an entire high school reported no improvement, or only minor improvement, on several academic indicators, such as graduation rate and college enrollment [7]. A third program reaching underserved minority students used community, school, and Internet-based resources to expose students to health careers. The program included a 2 week summer virtual science camp employing videoconferencing for real time demonstrations and interactions with health providers and webinars on test preparation, college admissions, and financial aid that supplemented in-person community, after school, and in school programs. Qualitative program evaluations indicated exposing students to a range of health professionals, involving parents and teachers, and offering long term interactive and ‘hands on’ learning experiences are effective strategies [8]. Finally, the National Library of Medicine has sponsored a program exposing minority high school students to a range of health professionals via a series of year long videoconferences. The program, which started as a small pilot with a single teacher at a single California high school [9], gradually expanded to include other teachers and classes and, eventually, additional schools in Alaska, Hawaii, and Puerto Rico [10]. Each presentation is rated by students and overall high ratings, comments by students and teachers during site visits, and program expansion are indicators of success.

Most career program research studies lack control groups and providing them can be an ethical issue if it means depriving certain students the potential benefits the programs are intended to provide [4]. It is important to know more than overall program satisfaction and preferences for particular methods and to determine program impact on career choice. Although it may be possible to determine college attendance and, possibly, initial majors, documenting them over time is extraordinarily difficult, involving extensive follow up with students throughout their post-secondary education. The problem is compounded because 48% of science, technology, engineering and math (STEM) students switch majors and about half change to non-STEM fields. Moreover, only 48% of STEM majors graduate [11]. Finally, health career programs for minorities lack a standardized curriculum. Most are predicated on the notion of exposing students to a range of health professions and professionals is beneficial so students understand they have more options than becoming a doctor or nurse and that there are many physician and nursing sub-specialties. In addition, attempts are made to involve professionals representative of the same minorities as the students who often tell very personal stories about their careers and career choices. The aim is to show that although health professions are demanding and require post-secondary education, they are attainable and have intrinsic, non-monetary rewards.

## Methods

This study assessed the potential effects of a career education program on career choice, by measuring career knowledge and interest over time. There was no control group due to ethical concerns and because the primary focus was to determine the effects of the program’s main components; on-site, in-person internship activities and follow up virtual distance mentoring.

The Research Center in Minority Institutions of the University of Puerto Rico Medical Campus has offered week long summer health science career internships for 13 years with funding provided by the National Institutes of Health, private foundations, and the university. Two internships are offered each year, one for high school and one for middle school students. Students are recruited annually from high and middle schools across Puerto Rico, representing all socioeconomic backgrounds. Students were recruited from three high schools (grades 10–12) and three middle schools (grades 7–9) for the study. The criteria for selecting these schools included their geographic distribution across the island (Northern, Eastern, Central, and Western regions of Puerto Rico), their access to internet service, and the identification of a liaison science teacher and an information technology person who could support the distance mentoring phase. The high school internship group had 25 students, 18 female and 7 male, while the middle school internship group had 24 students, 19 female and 5 male. Participating students and their parents signed a written consent explaining the study that was approved by the Institutional Review Board of the University of Puerto Rico.

During the week varied faculty conduct experiments demonstrating their research and students majoring in different health professions describe their educational experiences and career choices. In this intervention, distance mentoring was done the following academic year in nine monthly 1 h videoconferences with students in their schools involving additional health science faculty. The primary difference between the on campus and distance programs was that the on campus experience included hands on activities, often in special labs and facilities, which could not be provided at a distance.

The Kuder Career Interest Assessment® and a Health Career Knowledge and Interest Inventory were administered three times; before and after the campus internship and at the end of the distance mentoring period. In addition, students were asked to write a short essay at each of these times describing health science fields, their beliefs about working in these fields, and their reasons for considering a health science career. Kuder assessment, health science inventory, and essay scores were used to determine the intervention’s effects after the on-site, in-person activities and follow up distance virtual mentoring on career interests and knowledge of health sciences.

The Kuder assessment has 16 career clusters ranging from science, technology, engineering and math; health science; information technology; architecture and construction to agriculture, food and natural resources; hospitality and tourism; government and public administration; and finance. These career clusters are theoretically linked to personality traits. The assessment is online and measures interests by presenting objects and activities identified with certain clusters and having students indicate which they prefer first, second, and third. Students are asked, for example, if they would like to have a workbench and tools, figure out how much money they spent last month, or write short stories. The assessment is a standardized instrument that has been under development since 1938 and used by millions of middle and high school students and adults worldwide [12]. Like similar career assessment services, Kuder also offers extensive online career, college, and education resources. Kuder was selected for this study because, at the time, almost all of its resources were available in Spanish. Kuder results show ranking for different career categories relative to each other, but also rates each category as being of high, medium, or low interest. These ratings were given the respective values of three, two, and one for analysis.

The Health Science Knowledge and Interest Inventory was developed from a review of the literature on health science careers. It was devised specifically for the study because the Kuder assessment has very broad, general career comparisons and there was a need to measure more specific aspects of health career knowledge and interests. The 20 item instrument was generated by a member of the research team and revised based on team feedback until judged to have considerable face validity. The inventory asks students to rate themselves in relation to several statements about the health sciences and health careers on a seven point Likert scale ranging from ‘strongly disagree’ to ‘strongly agree’. Some statements asked them to judge their knowledge: ‘I have substantial knowledge of varied health careers’, ‘Interest and knowledge of science is beneficial in pursuing a health career’, ‘I am confident I can fulfill the education requirements for a career in a health field’. Other statements asked them to judge their attitudes: ‘I am definitely committed to pursuing a career in a health field’, ‘I could make substantial contributions to humanity by pursuing a health career’ and ‘I like working with people and helping them, even if they are sick, injured, or disabled’. Still other questions related to the nature of work in health professions: ‘I like working with equipment and technology’, ‘Healthcare providers have to be good listeners and communicators’, ‘Generally the higher level of education required for a health career, the higher the income’.

Students wrote essays about health professions before and after the on campus internship and at the end of the mentoring period. These were independently scored by two reviewers who were blind to the time essays were written. The reviewers compared scores for each essay and reached consensus. Essays were scored on four criteria that were assigned up to five points each, for a maximum possible score of 20 points. The first criterion was expansiveness or referencing a range of health careers, not just doctor or nurse. The second was referencing the nature of the work, including varied or long hours, teamwork, and the need for communication skills. The third was education level, including a range depending on occupation that could include an associate degree, professional degree, and/or graduate work. The fourth was personal traits and altruism, such as helping people, solving problems, and other non-monetary rewards in health science fields. These criteria reflect explicit and implicit goals of many health career programs.

To test for differences within groups across time, Wilcoxon non-parametric two-tailed exact tests were used. To test for differences between educational levels, non-parametric Mann-Whitney two-tailed exact tests were used. The alpha level for significance was set to .05 and calculations were made using the SPSS statistical package. Parametric tests were avoided because of the study’s sparse sample sizes would violate assumptions of normality.

## Results

Kuder assessment, health science knowledge and interest inventory, and essay scores pre internship, post internship and post mentoring are shown in Table 1. Kuder scores are broken down for the career clusters related to the health sciences and those related to science, technology, engineering and math (STEM). There were no significant (*p* < .05) changes in these scores over the three observation times for either measure and middle and high school student scores were not significantly different (*p* < .05). The mean health scores were significantly higher than STEM scores at pre internship (*p* = .0008), post internship (*p* = .003), and post distance mentoring (*p* = .005, Wilcoxon Signed Rank Exact Test).

**Table 1:** Assessment measure results and significance levels

		At Enrollment			At Post Internship			At Post Distance Mentorship			
Measures	School Level	*N* ^a^	Mean Scores & Standard Deviations	*p* values M*W*/Wilcoxon	*N* ^a^	Mean Scores & Standard Deviations	*p* values MW^d^/Wilcoxon	*N* ^a^	Scale Range	Mean Scores & Standard Deviations	*p* values MW^d^/Wilcoxon
Kuder Assessment – Health	Middle	25	2.6 ± 0.0.57	.09	22	2.64 ± 0.58	.54	19	1–3	2.63 ± 0.59	.66
	High	28	2.86 ± 0.35	–	19	2.74 ± 0.56	–	10		2.70 ± 0.67	–
	Average		2.74 ± 0.48	–		2.6 ± 0.56	.77			2.66 ± 0.61	19.99
Kuder Assessment – Science, Technology, Engineering, Math	Middle	25	2.20 ± 0.70	.58	24	2.23 ± 0.68	.99	17	1–3	2.05 ± 0.70	.75
	High	25	2.32 ± 0.61	–	25	2.26 ± 0.56	–	10		2.20 ± 0.42	–
	Average		2.26 ± 0.65	–		2.2 ± 0.6	.99			2.1 ± 0.6	23.27
Health Science Knowledge/Interest Inventory Education level	Middle	25	5.48 ± 0.56	.74	24	5.7 ± 0.44	.50	17	1–7	5.8 ± 0.5	0.04^c^
	High	25	5.52 ± 0.47	–	25	5.53 ± 0.49	–	10		5.5 ± 0.3	–
	Average		5.50 ± 0.51	–		5.61 ± 0.47^b^	.0.012^b^			5.72 ± 0.46^b^	0.007^b^, .074
Essay	Middle	25	8.36 ± 3.8	.54	18	10.22 ± 2.13	–	18	1–7	15.7 ± 0.5	.78
	High	28	7.43 ± 1.7	–	15	15.2 ± 2.8^b^	.0008^b^	15		15.6 ± 2.4	–
	Average		7.8 ± 2.9	–		12.4 ± 3.5^c^	.0001^b^			15.7 ± 1.8^c^	0.001^b^, .001

^a^ Number of students, ^b^Post internship and post mentoring means significantly higher than pre-internship, Wilcoxon Signed Rank Exact Test, ^c^Post mentoring mean significantly higher for middle school than high school students, Mann Whitney Exact Test. ^d^Post internship mean significantly higher for high school than middle school students, Mann Whitney Exact Test

Both the post internship and post distance mentoring Health Science Knowledge and Interest Inventory means were significant higher than the pre internship mean (*p* = .012, *p* = .007) and they were similar to each other, but approached being significantly different (*p* = .074). There were no significant differences between middle and high school student ratings pre internship (*p* = .75) or post internship (*p* = .50), but middle school student ratings were significantly higher than high school student ratings after mentoring (*p* = .04). Since the actual means (5.8118 and 5.5700) varied a little more than two tenths, this difference might be because far fewer high school student responded at the end of the mentoring period. Inventory reliability (Cronbach’s alpha) for the three administrations was .74, .74, and .68. Reliability was lower at the end of the mentoring period because fewer students completed the scale at that time. The lower number of respondents affected the reliability calculation.

The post internship and distance mentoring essay scores were significantly higher than those pre internship (*p* = .0001, *p* = .001). Post distance mentoring scores also were significantly higher than post internship scores (*p* = .0001), and there were no significant differences between middle and high school student essay scores pre internship and post mentoring (*p* < .05), but high school student essay scores were significantly higher than those of middle school students post internship (*p* = > .0008).

Additional analyses were performed on mean essay criteria sub scores by time of observation and education level and their significant differences that are not shown in Table 1. There were significant differences in scores for criteria 1 (expansiveness), 2 (nature of work), and 3 (education level) between the pre and post internship essays and between the pre and post distance mentoring essays (*p* > .05), but not for criterion 4 (altruism). High school means were significantly higher than middle school means post internship for the first 3 criteria (*p* > .05) but not post mentoring, where middle school means were significantly higher than high school post mentoring for criterion 1. High school post mentoring means were lower than post internship means for all criteria and may have been affected by low number of high school students completing the post mentoring essay. After mentoring, sub scores were only significantly higher for middle school students for criterion 1 (*p* > .05) and both groups scored the same for the remaining criteria.

## Discussion

There are four reasons that there may have been no significant shifts in Kuder scores over time. First, the assessment measures general interests that are theoretically related to personality traits that are more stable and unchangeable with age. Kuder research indicates career interest are mostly shaped during childhood and adolescence [13] and students may have formed solid career interests at the time of the internship. Second, the students voluntarily enrolled in the program and already had a high degree of interest in the health sciences. The average health science career rating, regardless of time of assessment was above 2.5 and the STEM average ratings were all above 2. Since the maximum possible rating was 3, the health science ratings especially, may have been subject to a ceiling effect. Initial ratings were high, leaving little room for improvement. Finally, attrition and non-compliance may have affected the outcome. The assessment takes time to complete and less than half the students taking it originally took it again at the end of the mentoring period. Finally, the Kuder assessment may not have been sufficiently specific and sensitive for measuring aspects of the program.

The Health Science Knowledge and Interest Inventory was shorter and specifically focused on aspects of health careers. Students consistently rated their knowledge and interest higher over time. Both the post internship and mentoring ratings were significantly higher than baseline before the internship and, although the post internship and post mentoring ratings were not significantly different from each other, they approached significance. High and middle school student ratings were about the same except after mentoring when middle school students rated their knowledge and interest significantly higher. Overall both groups felt that their knowledge and interest in health science careers improved at each stage of the program. Post internship mentoring may have benefited middle students more, perhaps because being younger they had less prior exposure to information about health careers.

Ratings of knowledge and interest were reinforced by essay scores which also significantly improved at each stage. Overall essay scores were significantly higher than baseline for both student groups post internship and post mentorship and overall post mentorship scores were significantly higher than post internship. There were significantly better scores over baseline for the first three scoring criteria after the internship and after mentoring. One reason criterion 4 (altruism) did not show significant improvement was that it had the highest baseline mean and was, perhaps, subject to ceiling effects. Although it shows students were more altruistically motivated to pursue health careers from the start, the mean for this criterion still improved by one point over baseline after mentoring. There were significant differences between high school student and middle school student essay scores for certain criteria at different assessment times. In some cases middle school students scored significantly higher and in others high school students did. More importantly, there were only significant differences between the groups on one criterion after mentoring and there were no significant differences in overall scores between high and middle school students by conclusion of the program. At the end of the mentoring period, the overall mean scores for both groups (15.60, 15.00 respectively) converged, indicating all students progressed about the same.

## Conclusion

Summer health career programs for middle and high schools students combined with distance mentoring can improve student knowledge, interest, and motivations for pursuing these careers, based on student self-reports and the quality of their essays. Short summer internship experiences had positive outcomes and follow up distance mentoring added value. The latter supports other programs using videoconferencing to provide career education virtually [8, 10]. Moreover, essay results largely support many of the explicit and implied aims of minority health science career programs; that they broaden student conceptions of what constitutes health careers, that students identify such careers are attainable despite their demands and educational requirements, and that such careers can be intrinsically rewarding. Whether the program can affect more general career assessments could not be determined based on this study’s findings. Indeed, the findings suggest that more global, standardized career assessments may not be sensitive to program goals.

One limitation of this study is that involved students who already expressed interest in health science careers which in some cases may have resulted in high pre-program baseline scores creating ceiling effects. Another limitation is that the study involves a single program offering and a small sample. Response rate may have affected results at the end of the mentoring period where there issues in getting high school students to complete the measures at the end of the academic year. Replicating the program and its evaluation would increase sample size and improve reliability. The study provides evidence, other than satisfaction, that health career education programs can work. Since the program had many of the same features of similar programs, especially exposure to a range of health professions, hands-on experiences, and online interaction, this study’s outcomes provide further evidence that these strategies are effective. Given the difficulties of following career student education and career paths after high school, the methodologies employed in this study might be employed along with satisfaction measures to determine whether other health career programs improve career knowledge and choice.

## Abbreviations

MW: Mann-Whitney Exact Test
STEM: Science, Technology, Engineering, and Math
